# Review: tunable nanophotonic metastructures

**DOI:** 10.1515/nanoph-2023-0034

**Published:** 2023-09-27

**Authors:** Yi-Chun Ling, Sung Joo Ben Yoo

**Affiliations:** Department of Electrical and Computer Engineering, University of California, Davis, CA 95616, USA

**Keywords:** nanophotonics, metastructures, metaphotonics, reconfigurable photonics, tunable photonics

## Abstract

Tunable nanophotonic metastructures offer new capabilities in computing, networking, and imaging by providing reconfigurability in computer interconnect topologies, new optical information processing capabilities, optical network switching, and image processing. Depending on the materials and the nanostructures employed in the nanophotonic metastructure devices, various tuning mechanisms can be employed. They include thermo-optical, electro-optical (e.g. Pockels and Kerr effects), magneto-optical, ionic-optical, piezo-optical, mechano-optical (deformation in MEMS or NEMS), and phase-change mechanisms. Such mechanisms can alter the real and/or imaginary parts of the optical susceptibility tensors, leading to tuning of the optical characteristics. In particular, tunable nanophotonic metastructures with relatively large tuning strengths (e.g. large changes in the refractive index) can lead to particularly useful device applications. This paper reviews various tunable nanophotonic metastructures’ tuning mechanisms, tuning characteristics, tuning speeds, and non-volatility. Among the reviewed tunable nanophotonic metastructures, some of the phase-change-mechanisms offer relatively large index change magnitude while offering non-volatility. In particular, Ge–Sb–Se–Te (GSST) and vanadium dioxide (VO_2_) materials are popular for this reason. Mechanically tunable nanophotonic metastructures offer relatively small changes in the optical losses while offering large index changes. Electro-optically tunable nanophotonic metastructures offer relatively fast tuning speeds while achieving relatively small index changes. Thermo-optically tunable nanophotonic metastructures offer nearly zero changes in optical losses while realizing modest changes in optical index at the expense of relatively large power consumption. Magneto-optically tunable nanophotonic metastructures offer non-reciprocal optical index changes that can be induced by changing the magnetic field strengths or directions. Tunable nanophotonic metastructures can find a very wide range of applications including imaging, computing, communications, and sensing. Practical commercial deployments of these technologies will require scalable, repeatable, and high-yield manufacturing. Most of these technology demonstrations required specialized nanofabrication tools such as e-beam lithography on relatively small fractional areas of semiconductor wafers, however, with advanced CMOS fabrication and heterogeneous integration techniques deployed for photonics, scalable and practical wafer-scale fabrication of tunable nanophotonic metastructures should be on the horizon, driven by strong interests from multiple application areas.

## 1 Introduction and background

Photonics plays an essential role in modern cyberinfrastructures across all aspects of computing, networking, and imaging. Our daily lives critically depend on sensing, processing, and communication of information. Global data traffic grew 11-fold over the past eight years (doubling every two years) [1] and the energy consumed on machine learning applications is now accelerating at the pace of doubling every 3.4 months [2]. With the Dennard’s scaling [3] already stalled since 2004 and the Moore’s Law [4] significantly slowing down as we aim nanometer-scale fabrication, progress in microelectronics and nanoelectronics alone cannot expect to achieve the required exponential improvements in energy-efficiency and throughput in the coming decades. Nanophotonics offers optical parallelism and energy-efficiency at the nanometer scale by taking complementary roles to nanoelectronics. Adding tunability to nanophotonics brings additional reconfigurable functionalities that can be valuable to many new applications. Reconfigurable nanophotonic technologies find applications in (a) photonic neuromorphic computing [5, 6], (b) optical interconnects in computing systems [7], (c) optical beam steering for light detection and ranging (LiDAR) and free-space-optical-communications [8, 9], (d) tunable optical filters [10, 11], (e) tunable lasers, and (f) computational imaging including compressive imaging and lensless imaging [12].

In terms of the construction of such tunable nanphotonics, there are (a) photonic crystal nanophotonics, (b) topological nanophotonics, (c) plasmonic nanostructures, and (d) metastructures. Such nanophotonic components constructed in waveguides or non-waveguide configurations achieve functionalities such as (a) tunable-optical-couplers for synaptic interconnects in optical neural networks [13, 14], (b) reconfigurable optical switches for reconfigurable computing, (c) tunable gratings for optical-beam-steering in LiDAR and free-space-optical-communications, (d) tunable optical resonance for tunable optical filtering, (e) tunable optical cavity in tunable lasers, and (f) reconfigurable metalens and metastructures for computational imaging including compressive imaging and lensless imaging.

Here, it is typically desirable for the tunable nanophotonic elements to achieve wide tuning ranges and high throughputs to enable more diverse functionalities at high throughput. For instance, broad-band operations in tunable optical filters and tunable lasers as well as near-hemispheric wide-angle beam-steering can be achieved when the nanophotonic devices can achieve relatively large changes in the optical index (Δ*n*) while keeping the optical absorption to be relatively small across the desired operating optical spectrum. Furthermore, some applications (such as neuromorphic computing and computational imaging) prefer such tuning can be non-volatile so that the reconfiguration can be set once without constantly maintaining such reconfiguration until resetting to a new configuration later, while some other applications (such as beam steering for LiDAR) need constant updates of reconfiguration states in order to address many different states quickly and dynamically. Examples of non-volatile reconfiguration mechanisms include (a) phase change of the materials (e.g. crystalline to amorphous and vice versa), (b) ferro-optical tuning, (c) ferro-magneto-optical tuning, and (d) mechanical tuning with latching (e.g. piezo-optical, MEMS, or NEMS tuning with latching). Examples of volatile reconfiguration mechanisms include (a) electro-optical tuning, (b) thermo-optical tuning, (c) magneto-optical tuning, and (d) non-latching mechanical tuning (MEMS or NEMS tuning without latching).

## 2 Tunable metastructure nanophotonics

Depending on the materials and the nanostructures employed in the nanophotonic devices, various tuning mechanisms can be employed. They include thermo-optical [15], electro-optical (e.g. Pockels and Kerr effects) [16–18], magneto-optical [19], ionic-optical [20], piezo-optical [21], mechano-optical (deformation in MEMS or NEMS) [22], and phase-change mechanisms [23–25]. In metastructures, it is possible to enhance the tuning effects thanks to the sub-wavelength structures [26]. This article will primarily review the tuning nanophotonics based on metastructures.

### 2.1 Phase-change-material tunable metastructure nanophotonics and applications

**Figure 1:**
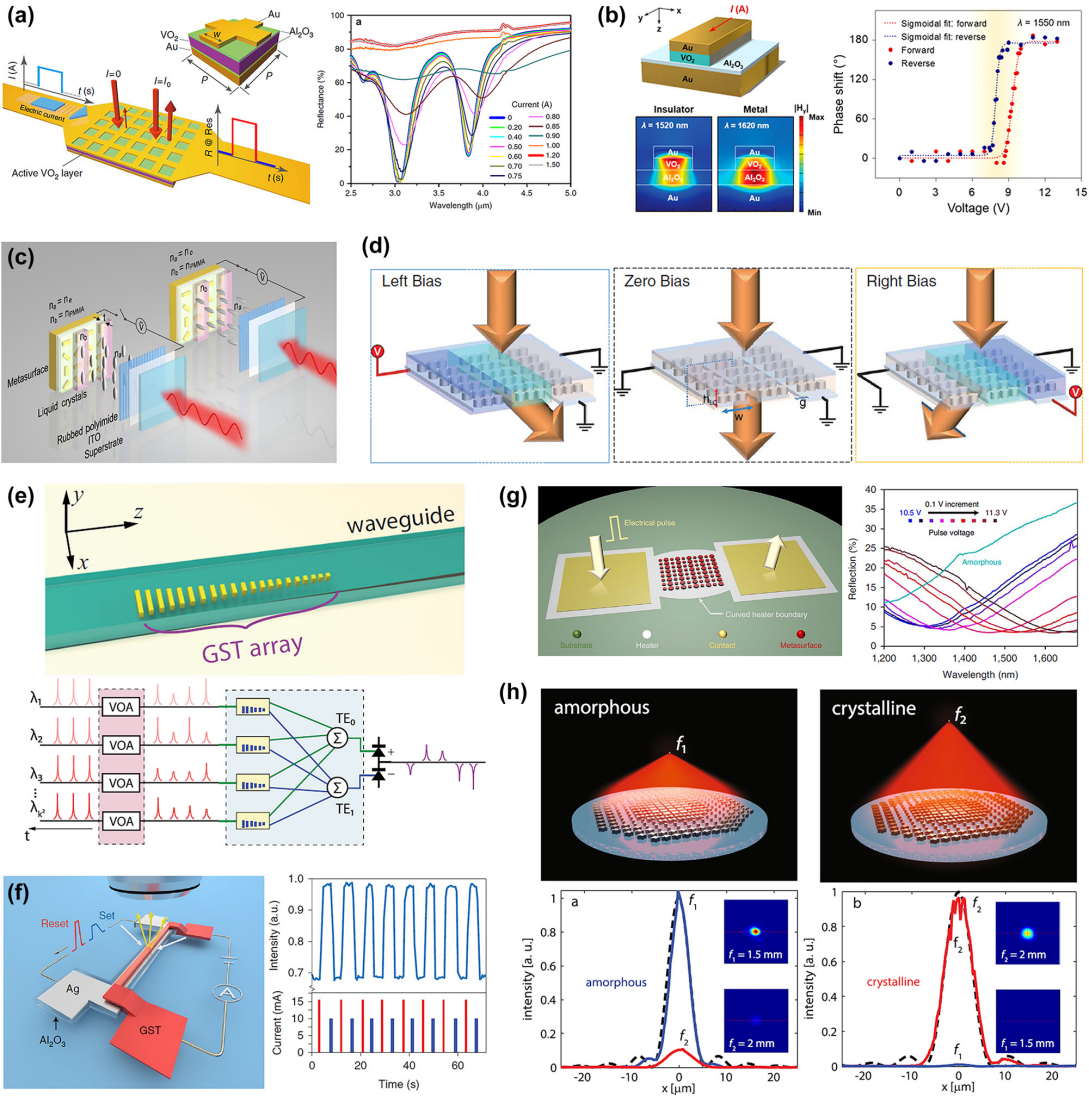
Tunable metastructures based on PCMs. (a) Schematic of a VO2-based sandwich metastructure and the corresponding measured reflection spectra under the different applied electric currents. Ref. [27] licensed under Creative Commons Attribution 4.0 License. (b) Schematic of a unit cell for a MIM structure incorporating a VO_2_ layer and the simulated magnetic field at the resonant wavelength under different VO_2_ phases. The curves illustrate the continuous phase shift as a function of the applied voltage. Reprinted (adapted) with permission from ref. [33]. Copyright 2019 American Chemical Society. (c) Schematic of a DMSD composed of a gold nanorod array and an LC layer to electrically control the light intensity. Ref. [36] licensed under Creative Commons Attribution 4.0 License. (d) Operation of an LC-based SLM integrated with a metasurface under different voltage bias. From ref. [37], reprinted with permission from AAAS. (e) Schematic of a phase-change mode converter to realize convolutional image processing. Ref. [38] licensed under Creative Commons Attribution 4.0 License. (f) Schematic and measurement results of programmable GST antennas operated by applying current pulses to transform GST between amorphous and crystalline states. Reproduced with permission from ref. [39]. Copyright 2021 Springer Nature. (g) Operation principle of an electrothermal GSST-based metasurface and the measured reflection spectra for the multi-state tuning with different voltages. Reproduced with permission from ref. [40]. Copyright 2021 Springer Nature. (h) Conceptual diagram of a varifocal GSST-based metalens and the corresponding measured focal spot profiles in amorphous and crystalline states. Ref. [41] licensed under Creative Commons Attribution 4.0 License.

Since vanadium dioxide (VO_2_) is able to significantly change electrical and optical properties during the phase transition, Werner et al. attempted to integrate a VO_2_ film into a gold metastructure to develop an electrically tunable platform for various nanophotonic systems, including electrically triggered information processing, storage, and display [27]. In their design, the metastructure is constructed by a sandwich structure of Au/Al_2_O_3_/VO_2_/Al_2_O_3_/Au, and a mesh pattern was applied at the top gold layer as illustrated in Figure 1(a). An electric current was applied to the top gold layer to investigate the performance of the electrical tuning, and the results show absolute amplitude tuning of 80 % and 75 % for the two absorber modes at 3.05 µm and 3.85 µm. Besides, a rectangular-shaped current pulse was utilized to examine the switching capability, and a rise and fall time of 0.2 s and 0.5 s were obtained, respectively. In addition, Kats et al. utilized defect engineering via ion beam irradiation to transform a VO_2_ film into a VO_2_ metasurface [28]. The ion beam irradiation on VO_2_ will cause lattice damage, resulting in the change of the critical temperature for VO_2_. By using ion beam irradiation through a mask, a VO_2_ metasurface with a specific pattern could be realized. The fabricated device exhibits a varying polarization-dependent reflection under different temperatures, which could be utilized in the development of tunable polarizers and absorbers. Schuller’s group further investigated the metal−insulator phase transition in VO_2_ and demonstrated a thermally tunable resonator which exhibits dielectric and plasmonic resonances under different temperatures [29]. Additionally, VO_2_ can be integrated with plasmonic metal nanostructures to create an active polarizer [30]. It is found that incorporating PCMs into polaritonic materials provides a means of manipulating polariton dispersion [31, 32]. Caldwell’s group demonstrated that the combination of hexagonal boron nitride (hBN) with VO_2_ can create a low-loss hyperbolic mestasurface and the corresponding wavelength tuning range for hyperbolic phonon polaritons (HPhPs) can be increased by a factor of 1.6. To achieve further scalability of this device, sputtering and metal organic chemical vapor deposition can be utilized to grow VO_2_ and boron nitride separately. However, the growth of large-area, high-quality hBN remains a challenging task. To experimentally demonstrate a wide phase modulation, Atwater et al. developed a reflectarray metasurface by employing a VO_2_ layer into a metal−insulator−metal (MIM) structure as indicated in Figure 1(b) [33]. The phase transition of VO_2_ via localized heating was found to enhance the magnetic dipole resonance in the metasurface, and a maximum phase shift of 250° for the reflected light can be achieved. The response time measurement showed the ON and OFF switching times of ∼15 ms and ∼100 ms, respectively. These promising results demonstrate significant potential in facilitating the development of beam steering systems, reconfigurable lenses, and holographic imaging. Although metal-based VO_2_ metasurfaces have shown various tunable functionalities, the efficiencies are still often limited by absorption loss, hindering the development of certain applications like optical communication and imaging. An all-dielectric Si–VO_2_ metasurface was demonstrated by Cueff’s group [34]. Since several recent works have shown that Mie resonant modes excited in nanostructures can assist the manipulation of optical features, VO_2_ film was incorporated into this design to enhance Mie resonance. The metasurface is composed of a Si nanodisk array on a 25-nm VO_2_ layer operating at ∼1.4 μm wavelength. The measured extinction spectra with different temperatures exhibit a significant magnitude tuning and a spectral tuning with almost ∼100 % absorption. Moreover, the hysteresis behavior was observed during the heating-cooling cycle, which may benefit the non-volatile memory applications. Since most previous works have focused only on the resonant modes of VO_2_-based metasurfaces in the near-infrared range, Šikola’s group conducted further investigations on the Mie resonance present in the visible spectrum [35]. The measurement results show significant modulation depths of 8 dB for scattering and 2.5 dB for extinction in the visible spectrum, highlighting the potential of creating tunable metasurfaces operating in this range.

In order to demonstrate the potential of practical applications using metasurfaces, active controlling has become one of the essential features to assess the performance. Kivshar et al. presented a silicon nanodisk metasurface integrated with a liquid crystal (LC) and investigated the dynamic tuning of electric and magnetic resonances [42]. The measurement results show a maximal resonance tuning of 40 nm and a transmission contrast of ∼500 %. The performance of LC-based metasurfaces can be limited by overly strong surface anchoring of LC molecules, which can hinder their spectral tunability. To address this critical issue, Fedotov’s group proposed a design that integrates plasmonic resonators with twisted LC cells, effectively eliminating the problem [43]. Their experimental results demonstrate a spectral tuning range of up to 110 nm under a voltage range of 1.5–2.7 V, paving the way for improved dynamic manipulation of optical wavefronts. LC tunability can also be used for beam deflectors. For example, Neshev et al. exploited a silicon nanodisk metasurface with LC to demonstrate the dynamic steering of a laser beam [44]. The proposed metasurface is composed of nanodisks with six different radii, resulting in a phase gradient profile on the metasurface. The deflection angle of the laser beam can be switched from 0° to 12° when the applied temperature on LC is increased from 25 °C to 60 °C and the transmission efficiency can reach 50 %. Of particular significance, the LC layer in this device has a thickness of only 130 nm, facilitating the development of ultrathin beam steering devices. To further advance the manipulation of the optical responses in metasurfaces, Liu et al. proposed and demonstrated a light project technology by tailoring the spatial frequency pattern [36]. In Figure 1(c), the proposed digital metasurface device (DMSD) is composed of a gold nanorod array and each nanorod has an orientation that can induce different geometric phase shifts. After employing an LC layer on the top of the gold nanorods, an extra propagation phase shift can be modulated with an applied voltage bias. Different total phase shifts will result in either propagation wave or evanescent wave corresponding to on or off states in each metasurface pixel, respectively. The intensity modulation ratio can reach up to 105:1, and the switching time for on and off states is 40 and 65 ms, respectively. Additionally, LC is often utilized in spatial light modulators (SLMs) due to its high birefringence, but the large pixel size for LC-based SLMs still limits the performance for certain applications like beam steering and imaging. To overcome this obstacle, Kuznetsov’s group integrated a TiO_2_ metasurface into an LC-based SLM to miniaturize the pixel size and realize phase-only modulation as shown in Figure 1(d) [37]. Incorporating a metasurface significantly reduces the required thickness of the LC layer to fulfill 2*π* phase modulation. The demonstrated SLM exhibits a maximal deflection angle of 11° with 36 % efficiency while the LC layer thickness requires only 1.5 µm and the pixel size is ∼1 µm, resulting in a more compact design than the traditional SLMs. To further improve the switching efficiencies and small angular deflection, Miller’s group used adjoint optimization-based inverse design to significantly enhance the device performance [45]. The optimized results can achieve tunable deflection angles ranging from 12° to 144°, with switching efficiencies above 80 %. As LC infiltration has emerged as a promising approach for tuning the optical properties of dielectric devices, Staude’s group designed a dielectric metasurface infiltrated with liquid crystals to realize an electrically tunable display in the visible spectrum [46]. Specifically, they employed the photoalignment technique for LCs to achieve outstanding homogeneity of the LC prealignment over the entire 20 × 20 mm^2^ area. Besides, Shvets’s group demonstrated a bifocal metalens by encapsulating amorphous silicon nanopillars in a nematic LC [47]. To overcome the challenges associated with scaling the size of the metalens, this device utilized only three types of meta-atoms. The experimental results demonstrate a significant focal length switch between 9 mm and 4.5 mm, achieved through a low voltage bias of just 9.8 V. The integration of metasurfaces with LC can expand the ability to manipulate light polarization. Duan et al. proposed and demonstrated a polarization-dependent LC-integrated metasurface by utilizing a new packaging method [48]. The geometric phase of the metasurface and the birefringence of LCs allow multiple applications including the switchable metaholograms and dynamic varifocal metalenses.

The nonvolatility and low-loss properties of the chalcogenide PCMs provide a great potential to develop high-performance optical computing. Given GeSbTe (GST) has remarkable features in the mid-infrared range [49, 50], it holds promise for advancing thermal imaging technology. To this end, Giessen’s group integrated an aluminum nanoantenna array with GST to create a tunable mid-infrared absorber [51]. The phase transition of GST from amorphous to crystalline states leads to a noticeable contrast in reflection and a shift in resonance. Notably, a demonstration was conducted using a superpixel arrangement comprising four distinct metasurface designs to achieve a temperature-selective absorber within the 2.5–4 μm wavelength range. Zheludev’s group has shown that a metasurface based on GST can also display exceptional resonance shift and switching contrast in the near-infrared spectrum [52]. Several GST-based hybrid metasurfaces have been proposed for their remarkable tuning capability in the near-infrared spectrum, including high modulation depth (70 %) [53] and phase tuning (>230°) [54]. In order to expand the operating bandwidth, Lee’s group introduced a U-shaped GST nanoantenna array that can operate over a range of 500 nm in the near-infrared range [55]. The development of tunable optical devices relies heavily on the capability of rapid modulation. In pursuit of this goal, Simpson’s group devised a heat-transfer model to derive the heat generation and temporal temperature distribution within the GST-based metasurface [56]. The calculated results indicate the reversible switching between two GST states can reach up to 300 ns. Given the substantial time and effort typically required to optimize meta-atom structures, Zhang’s group has adopted a deep learning modeling approach to improve the efficiency of characterizing metasurfaces [57]. The deep neural network (DNN) approach allows for the derivation of amplitude and phase responses induced by 3D meta-atoms in millisecond time scale and facilitates the creation of inverse designs for complex multifunctional metasurfaces. In Figure 1(e), Li et al. reported a programmable waveguide mode converter using GST-based metasurface [38]. The significant refractive index change during the GST phase transition was utilized to control the waveguide spatial modes, resulting in a high modal contrast level. This contrast was subsequently applied in a neural network to perform the image processing and recognition tasks under the computation speed of ∼1 kHz. A key contribution of this work is the presentation of a 2 × 2 array prototype system that can be easily scaled up to a larger network using a photonic crossbar array architecture. However, optical loss becomes a significant concern in a large network, particularly as on-chip optical amplification has not yet been fully developed. Additionally, the intermediate GST phases can be employed in metasurfaces to realize tunable filtering. Kim et al. designed a 1-inch diameter metasurface with a GST nanohole array and demonstrated a narrowband filtering at the wavelength range of 3–5 µm [58]. A pulsed laser with nanosecond duration was applied in the proposed metasurface to shift the resonance in spectra and the switching is reversible. A passband of ∼74 nm can be achieved while the transmittance of ∼70 % can be maintained. This proposed design demonstrates remarkable scalability, as it can be easily scaled up to larger dimensions through the use of widely adopted industry-standard UV-lithographic and physical vapor deposition techniques. While much research has focused on the significant differences between the amorphous and crystalline states of GST, Lee’s group has introduced a novel hybrid state that represents a co-existing mixture of amorphous and crystalline GST-based meta-atoms [59]. In addition, a novel holographic technique has been developed to visualize information that is only discernible in the hybrid state GST metasurface. This novel approach has the potential for significant applications in fields such as all-optical image encryption, security, and anti-counterfeiting. In addition to optical switching with laser pulses, electrical tuning is another common way to induce phase transitions in PCMs. As presented in Figure 1(f), Brongersma’s group put silver strips under a GST antenna array to construct nano-heaters for each element and excite stronger resonance between the GST and silver layers [39]. Such a heater design can make the heat dissipate to the substrate faster and speed up the cooling process, which can shorten the switching time between the amorphous and crystalline states. In this work, the operation speed can reach the 10 kHz range and the reflectance modulation can achieve 30 % at the wavelength of 730 nm. Later, Adibi’s team made a significant advancement by developing a chip-scale metadevice that enhances microheater design and achieves higher level of optical efficiency [60]. Instead of using the plasmonic materials like silver for the microheater [39], Tungsten was chosen in this work thanks to its exceptional combination of high melting point, excellent thermal conductivity, moderate resistivity, and low thermally activated diffusion. Furthermore, an Al_2_O_3_ layer and a HfO_2_ layer were utilized to facilitate heat exchange between the microheater and the metasurface, and preserve the generated heat for the metasurface, respectively. In terms of performance, this metadevice can achieve a remarkable 80 % absolute reflectance contrast, with the ability of spectral tuning over 250 nm. Instead of heating the entire PCM-based metasurface for optical tuning, Zheludev et al. applied femtosecond laser pulses on a small area of GST to induce phase transition [61]. Different patterns can be written and erased by laser pulses multiple times and the smallest hotspot can reach the sub-diffraction limit of 0.49 μm under the wavelength of 730 nm. By using this non-volatile universal reconfigurable platform, several applications including focusing, hologram, and resonance tuning can berealized. Besides, Li’s group has shown that laser direct writing techniques can be used to encode both infrared and visible patterns into a GST layer [62]. This breakthrough paves the way for potential applications such as IR data storage, encryption, and camouflage. A new PCM Ge–Sb–Se–Te (GSST) with broadband transparency at 1–18.5 μm was reported by Hu et al. [63]. Similar to GST, GSST also shows a large refractive index change during the phase transition and the wide transparency window can further expand the operational wavelength of PCM-based photonic devices from near-infrared (NIR) to long-wave infrared (LWIR). A non-volatile electrothermal switching with record low loss was demonstrated, and the amorphization switching time can reach a microsecond. These results pave the way for performance improvement of PCM-based tunable metasurfaces, and the significant change in refractive index makes it possible to further downscale metadevices, In order to showcase the ability of GSST to be integrated with various photonic platforms, Hu’s group developed an electro-thermal switching technique for GSST-based metasurfaces, which utilized a single-layer graphene microheater [64]. By applying different sequences of electric pulses, they were able to identify four distinct levels of crystallization, achieving an impressively low power consumption of only ∼8.6 mW. However, a current saturation phenomenon was observed due to surface polar phonon scattering, which impacts the power supply of the system and requires further investigation. Furthermore, they created a 3D model to evaluate the performance of a GSST-based metasurface integrated with a graphene microheater. The results showed that optical phase tuning of up to 294° was achievable, indicating a promising new approach for developing active metasurfaces. To enable computationally efficient photonic design, Hu’s group combined DNNs with analytical optimization based on the transfer matrix method (TMM) [65]. By using this approach, they successfully predicted and inverse designed a tunable mid-wave infrared bandpass filter comprising two distributed Bragg reflectors (DBRs) and one metasurface layer in between. These results indicate that this TMM approach used in this study can be applied to diverse hybrid design schemes such as waveguide devices, photonic crystals, and stacked multilayer metasurfaces. To scale up PCM-based optical devices, it is essential to maintain uniform electrothermal switching across large areas. In Figure 1(g), a large-scale reconfigurable GSST-based metasurface with optimized heaters was reported by Hu et al. [40]. The voltage control was exploited to realize multi-state tuning of PCM and record half-octave spectral tuning was obtained with the optical contrast up to 400 %. Although the intermediate phases of PCM have been utilized for continuous tuning of optical properties, the phase precision needs to be further addressed and quantified. As shown in Figure 1(h), Gu et al. proposed a generic design methodology to realize arbitrary phase profiles for metasurfaces and a bi-state varifocal metalens was demonstrated as a proof-of-concept [41]. The GSST-based Huygens’ meta-atoms are used in this design, and 16 meta-atoms were chosen to realize four-level discretized phase profiles. The measured images exhibit an aberration-free diffraction-limited focusing at 5.2 μm wavelength with low crosstalk and the switching contrast ratio can reach 29.5 dB.

### 2.2 MEMS/NEMS tunable metastructure nanophotonics and applications

**Figure 2:**
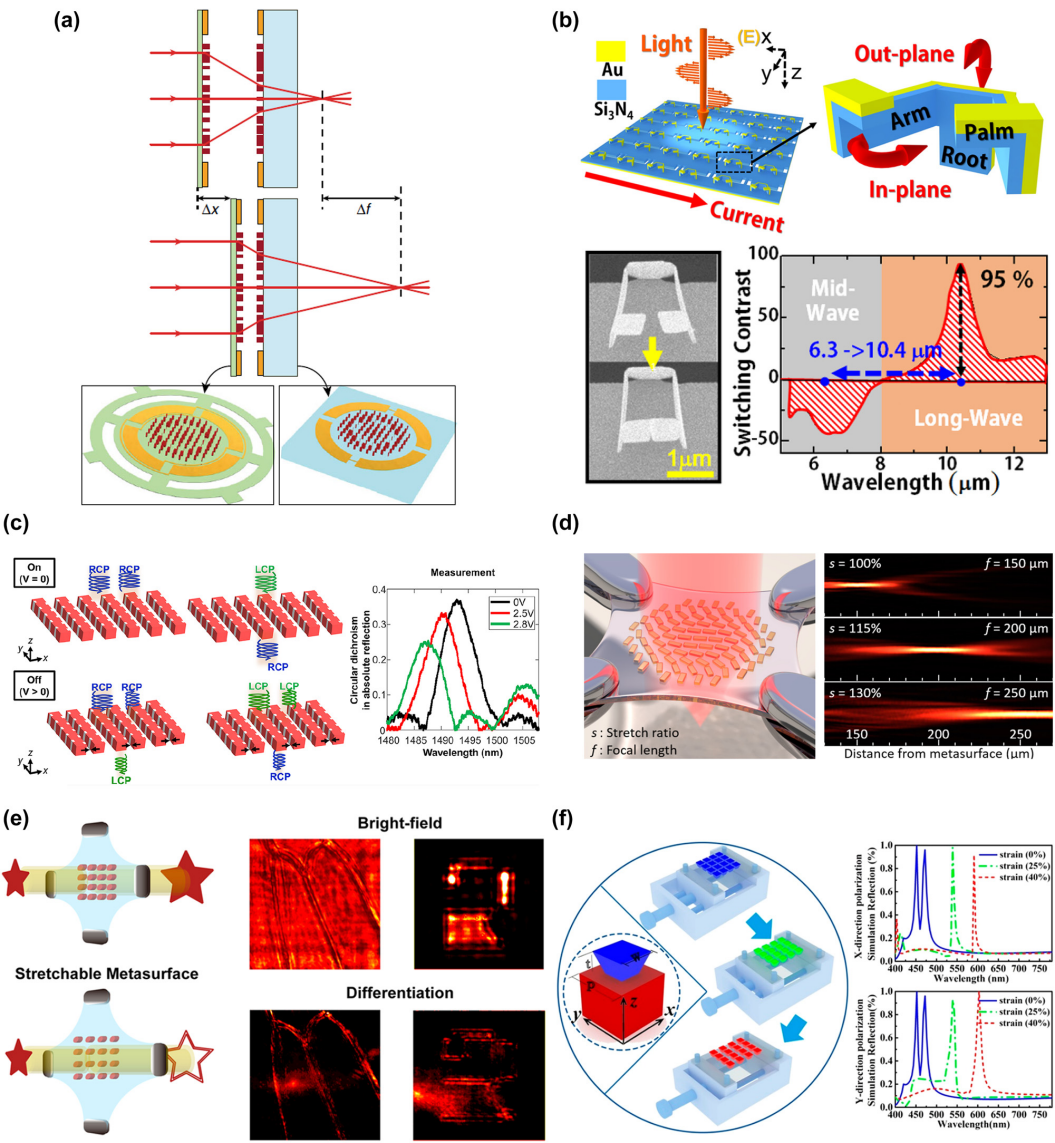
Tunable metastructures based on mechanical systems. (a) Schematic of a metalens doublet composed of a stationary metalens and a moving metalens. Ref. [66] licensed under Creative Commons Attribution 4.0 License. (b) Working principle of a nano-SRR array deformed by electro-thermo-force and the corresponding switching contrast as a function of the wavelength. Reprinted (adapted) with permission from ref. [67]. Copyright 2016 American Chemical Society. (c) Schematic of a nanoelectromechanically tunable chiral metasurface with different chiroptical effects and the measured circular dichroism under different applied voltages. Reprinted (adapted) with permission from ref. [68]. Copyright 2021 American Chemical Society. (d) Schematic of a metastructure on a stretchable PDMS substrate and the measured beam profiles along longitudinal direction under different stretch ratios. Reprinted (adapted) with permission from ref. [69]. Copyright 2016 American Chemical Society. (e) Schematic illustration of a PDMS-based metasurface under different strains and the corresponding measured images for bright-field and differentiation. Reprinted (adapted) with permission from ref. [70]. Copyright 2021 American Chemical Society. (f) Schematic and simulated reflection spectra of a polarization-insensitive metastructure composed of TiO_2_ nanoblocks on a PDMS substrate. Reprinted (adapted) with permission from ref. [71]. Copyright 2020 American Chemical Society.

The techniques of microelectromechanical systems (MEMS) and nanoelectromechanical systems (NEMS) have been widely used for the infrared and terahertz regions. To integrate MEMS into tunable metasurfaces, Faraon et al. designed and fabricated a metasurface doublet with an electrically tunable distance between them [66]. As shown in Figure 2(a), the metasurface doublet is composed of a stationary diverging metalens on a glass substrate and a moving converging metalens on a SiN_x_ membrane. A large optical power change of 60 diopters can be achieved while the SiN_x_ membrane is actuated to move only a 1-μm distance. The frequency response measurement shows the first two mechanical resonance modes at ∼2.6 and ∼5.6 kHz, limiting the maximum scanning frequency for this system to ∼4 kHz. Since most of the mechanical manipulations for metasurfaces are limited to only one direction, Wu et al. designed a nano split-ring-resonator (SRR) array which can be tuned in both in-plane and out-plane directions [67]. In Figure 2(b), the proposed SRR is a composite of gold and silicon nitride and the structure is composed of a root and two movable arms with palms. When a current is applied to the device, the gap width between the palms can be changed from 514 nm to 0 nm, and the root part can have a deformation of 17.4° bending. These two variations lead to in-plane and out-plane rotations for the SRRs, respectively, and the process is reversible. The measured reflection spectra show that modulation depth can reach up to 95 % and the resonance wavelength can be switched between ∼10 μm and ∼6 μm at the ON and OFF states. The response time is estimated to be 20–30 μs based on the conduction and cooling time, corresponding to a potential modulation speed of ∼50 kHz. Besides, tunable metasurfaces can be utilized for circular polarization filtering. Figure 2(c) shows that Faraon et al. designed and demonstrated a dielectric chiral metasurface that can be tuned electromechanically [68]. The metasurface was fabricated on a silicon-on-insulator (SOI) wafer, and it is composed of two sets of doped silicon nanostructures with gold electrodes deposited on each side. When a bias voltage is applied to the electrodes, an electrostatic force between the silicon structures will be induced, resulting in the variation of the gap width. The experimental results show that the reflective circular dichroism (CD) is modulated from 0.45 to 0.01 when the voltage is increased from 0 to 2.75 V. The significant change of CD exhibits the capability of circular polarization filtering, and the low operating voltage can be beneficial for on-chip integration. However, the modulation speed is limited to only 100 Hz attributed to the low doping density of the silicon layer in the SOI wafer.

Utilizing stretchable substrates is also a popular method to realize mechanical tuning of photonic devices due to simpler fabrication processes. Agarwal et al. demonstrated a metasurface on a stretchable polydimethylsiloxane (PDMS) substrate, and the optical properties can be manipulated with different stretching ratios as indicated in Figure 2(d) [69]. The proposed metasurface consists of a gold nanorod array, and the PDMS substrate can be stretched by four clamps to change the lattice constant of the nanorods. By using this method, a PDMS-based metasurface was designed for anomalous refraction, and the results exhibit an adjustable refraction angle from 11.4° to 14.9° when the substrate is stretched by ∼30 %. Another design for a zoom lens was presented and showed a continuous tuning of focal length from 150 nm to 250 nm at the wavelength of 632.8 nm. Besides, PDMS-based metasurfaces can be used to modulate the spatial frequency of images as shown in Figure 2(e) [70]. When the lattice period changes with different strains, the coupling properties between meta-atoms change, resulting in different Green’s functions performed by a metasurface. Based on this mechanism, Valentine’s group proposed and demonstrated a metasurface that can switch the imaging between bright field and 2D differentiation under different strains, and the operation bandwidth can achieve 60 nm. However, if the applying strains along the two orthogonal linear polarization are not identical, the stretchable metasurfaces will appear with anisotropic optical properties. To overcome this problem, Xiao et al. employed a TiO_2_ metasurface on the PDMS substrate and studied the resonance properties of electric dipoles and magnetic dipoles in the TiO_2_ metasurface [71]. In Figure 2(f), the experimental results show identical reflection spectra in the visible region for the two orthogonal linear polarizations under different strains, which can be regarded as the polarization-insensitive response for the stretchable TiO_2_ metasurface.

### 2.3 Electro-optically tunable metastructure nanophotonics and applications

**Figure 3:**
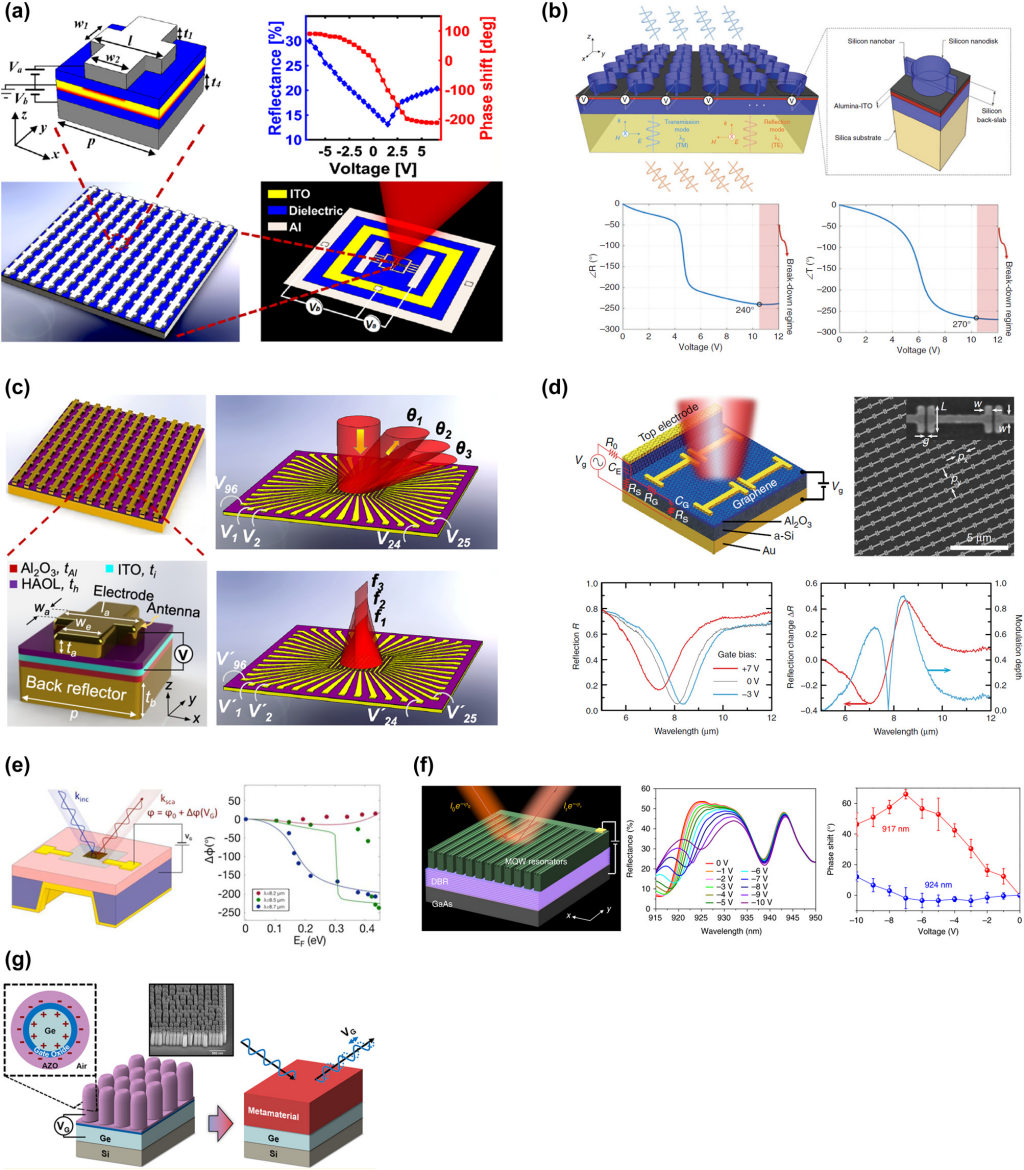
Tunable metastructures based on electro-optical modulation. (a) Schematic of a dual-gated metasurface embedded with an LTO layer and the measured reflectance and phase shift as a function of applied voltage. Reprinted (adapted) with permission from ref. [76]. Copyright 2018 American Chemical Society. (b) Schematic of an electrically tunable metasurface with SIS formation and the calculated phase of reflected/transmitted light versus applied voltage. Ref. [79] licensed under Creative Commons Attribution 4.0 License. (c) The schematic of a multifunctional ITO-based metasurface that allows the applications of beam steering and dynamic light focusing. Reprinted (adapted) with permission from ref. [80]. Copyright 2020 American Chemical Society. (d) Schematic and SEM image of a hybrid graphene metasurface operating in the mid-infrared and the properties of the reflected light under different gate biases. Ref. [81] licensed under Creative Commons Attribution 4.0 License. (e) Schematic illustration of a graphene-gold metasurface operated by applying a gate voltage and the corresponding phase modulation versus the Fermi energy of the graphene. Reprinted (adapted) with permission from ref. [82]. Copyright 2017 American Chemical Society. (f) Schematic of an MQW-based metastructure and the measured reflection spectra and phase shift under different applied bias voltages. Ref. [83] licensed under Creative Commons Attribution 4.0 License. (g) Schematic and an SEM image of Ge nanopillars coated with a gate oxide and AZO to form SOS capacitors. Reprinted (adapted) with permission from ref. [84]. Copyright 2018 American Chemical Society.

Indium tin oxide (ITO) is one of the representative active materials for electro-optical modulation. Brongersma’s group integrated a gold strip array with ITO to create MIM plasmonic cavities, resulting in the realization of an active absorber [72]. Morevover, this study has demonstrated that operating ITO within the spectral range of epsilon-near-zero (ENZ) can lead to a significant modulation of absorption. Additionally, Atwater’s team designed an active polarization converter that leverages the ENZ region of ITO [73]. This study involved the integration of an aluminum nanoantenna array with an ITO layer to create the proposed metasurface. To modulate the refractive index under the ENZ condition, a voltage bias was applied between the ITO layer and the back reflector. Their simulations indicate that the proposed device has the ability to convert linearly polarized light into various polarization states of the reflected light, such as cross-polarized, circularly polarized, or elliptically polarized light. Atwater’s group has advanced the tunable capabilities of metasurfaces by demonstrating an electrically gate-tunable metasurface [74]. The proposed metasurface design consists of a gold backplane, an ITO layer, and an aluminum oxide layer with a gold stripe antenna array on top. Field-effect modulation was employed to tune the complex refractive index of this structure, resulting in a 180° phase shift and a 30 % reflection contrast under 2.5 V of gate bias. By applying bias to specific subgroups of metasurface elements, they were able to demonstrate the switching of ±1 order diffracted beams, highlighting the potential for beam steering devices. Additionally, Atwater’s group demonstrated an ITO-based metasurface with an extremely low modulation voltage by utilizing a novel modulation mechanism [75]. The proposed metasurface design includes nanostructured silver and ITO electrodes separated by a 5 nm thick alumina layer. The study found that applying bias causes silver ions to transport through the alumina layer, resulting in the nucleation and growth of silver nanoparticles in the ITO counter-electrode. Thanks to this novel mechanism, they were able to achieve a reflectance change of 30 % with a bias of only 5 mV, indicating potential for the development of attojoule-energy scale optical modulators. In Figure 3(a), Atwater et al. reported a significant phase tuning at 1550 nm by using a dual-gated metasurface [76]. In the proposed architecture, an ITO layer was embedded between the two gate dielectrics and an aluminum fishbone array was placed on the top, which can be considered as a two series-connected MOS field effect structure. The measurement results show a large continuous phase shifting up to 303° and a reflection contrast of 89 %, which can be leveraged for various applications such as varifocal lenses, dynamic holograms, and beam steering devices. Similarly, Mosallaei’s group proposed another dual-gated ITO structure integrated with guided-mode resonance mirror (GMRM) which incorporates a Si nanograting and a Si guiding core [77]. This device can achieve a modulation depth of up to 0.8 and a phase modulation of 210° with an applied voltage bias ranging from −15 V to 24 V. In addition, Mosallaei’s group incorporated a multigate biasing technique into an ITO-based metal-insulator-semiconductor (MIS) structure to minimize dissipative loss at resonance [78]. With this approach, they presented a phase modulation of 180° while maintaining a reflection of 0.4 in the near-infrared range. ITO-based metastructures are usually performed in reflection mode, which may hinder the integration capability with other photonic devices. Therefore, Mosallaei et al. proposed a semiconductor-insulator-semiconductor (SIS) structure hybridized with ITO to achieve tunable dual-mode modulation [79]. In Figure 3(b), the SIS structure is constructed by combining Si nanostructures, an ITO-alumina interface, and a Si slab. In order to realize the dual-mode operation, the shape of the nanostructures was designed to be a mixture of nanodisks and nanobars, which can excite the optical resonances for the reflection and transmission modes separately. By applying a bias voltage between the Si nanostructures and the ITO layer, Mosallaei’s group achieved a 240° phase shift for the reflected transverse-electric (TE) polarized light and a 270° phase shift for the transmitted transverse-magnetic (TM) polarized light. In the past, topology optimization has typically been used for designing passive metasurfaces, but this approach can limit the versatility of the device. Therefore, Atwater’s group presented an array-level inverse design approach to create active metasurefaces [8]. This method allows for the easy realization of nonintuitive designs that can provide promising features, even in cases where some meta-atoms have nonidealities. In this work, they were able to increase the directivity of antenna arrays to 84 %, and experimentally demonstrated a phase modulation of approximately 220°. Although tunable metasurfaces can be modulated by the above mechanisms, it is still challenging to create a reconfigurable arbitrary phase profile of a metasurface. As a proof of concept, Atwater’s group designed and demonstrated a multifunctional ITO-based metasurface by controlling the optical phase shift of each metasurface element individually [80]. Since the refractive index change in the ITO layer can result in a phase shift higher than 270°, the phase shift value was discretized into four levels (0, 90°, 180°, and 270°) to realize an arbitrary phase profile. This multifunctional metasurface was demonstrated with dynamic beam steering and light focusing as shown in Figure 3(c). It is noted that the performance of this device was presented in only one dimension. Thus, the architecture of the metasurface must be further improved to carry out more optical functions that require two-dimensional optical phase control.

Graphene has shown extraordinary features in electrical and optical response. For example, Zhang’s research team introduced a plasmonic metastructure that utilizes a perforated graphene layer with quadrupole slot structures and dolmen-like slot structures to manipulate transparency windows [85]. In this study, they found that the graphene-based quadrupole slot structure displays a single transparency window, whereas the dolmen-like slot structure exhibits two transparency windows due to an extra transmission dip. Furthermore, we can vary the Fermi energy levels of the graphene layer to shift these transparency windows, thereby creating new opportunities for designing tunable multiple-wavelength slow light devices, plasmonic switches, and optical sensors. Likewise, Zhao’s group proposed a different plasmonic metamaterial structure comprising of three graphene layers interwoven with a silicon grating, and by varying the Fermi energy of the graphene layers, they were able to tune the dual plasmon-induced transparency peaks [86]. Since most previous work on graphene has focused on the optical features of fundamental or low-order modes, Tian’s research team numerically demonstrate a gradient graphene metasurface to manipulate the wavefront of high-order modes [87]. In this work, they were able to achieve high-order anomalous reflection modes with a significant phase shift exceeding 2*π* and ultra-high-order vortex beams up to 15 orders, which have potential applications in beam splitters and high-NA plasmonic lenses. In addition, graphene is frequently incorporated into metasurfaces to create perfect absorbers [88, 89]. For example, Capasso’s research team proposed an electrically tunable perfect absorber by integrating graphene into a metasurface and an asymmetric Fabry−Perot resonator [88]. By applying a gate voltage on graphene, they achieve a modulation depth nearly 100 % and the modulation speed can reach up to 20 GHz at the wavelength range of 5–7 μm. Furthermore, Atwater’s team employed a combination of low permittivity substrate and plasmonic metallic antennas to enhance the radiative coupling with graphene plasmonic ribbons, leading to a remarkable modulation depth of 95.9 % in reflection [89]. Some research has attempted to apply graphene in metasurfaces to realize more specific applications in the infrared. For example, Chen et al. demonstrated a hybrid graphene metasurface operating in the mid-infrared range as indicated in Figure 3(d) [81]. A record high modulation speed of ∼1 GHz was achieved while a low gate voltage bias between –3 V and 7 V was applied. Then this hybrid graphene metasurface was incorporated into a prototype spatial light modulator (SLM) for the demonstration of single-pixel imaging. The experiment results reveal better imaging speed compared to that of traditional LC-SLMs. Besides, graphene metasurfaces can be employed for optical phase control in the mid-infrared [82]. In Figure 3(e), Atwater’s group presented a graphene-gold metasurface and the optical phase of the reflected light can be modulated by tuning the gate voltage between the graphene and the gold reflector. Since the Fermi energy of graphene changes with different gate voltages, the corresponding permittivity can be modulated at the same time, which results in the optical phase shift of the reflected light. The experimental results indicate that the phase shift can reach up to 237° at the wavelength of 8.5 μm and the bandwidth was found to be over 250 nm. Similar to Zhang’s aforementioned study, Shi’s group proposed a metasurface composed of two graphene nanodisks and a graphene nanostrip, resulting in a dipole resonance and a quadrupole resonance respectively [90]. The corresponding symmetric resonance mode and asymmetric resonance mode create a transparency window in mid-infrared range. Furthermore, they evaluated the effectiveness of this metastructure as a refractive index sensor, and the simulation results indicate that it can achieve a sensitivity of 3016.7 nm/(RIU) and a figure of merit (FOM) greater than 12.0. While numerous proposed active metasurfaces demonstrate exceptional phase modulation, simultaneous control of amplitude remains a challenge, leading to suboptimal reconstruction of the intended wavefronts. In order to address this issue, Jang’s team proposed a metamolecule design composed of a pair of gold antennas, each in contact with graphene plasmonic ribbons (GPRs) [91]. By allowing for individual control of the Fermi energy for the two constituent graphene metaatoms, it becomes possible to achieve complete control over both the amplitude and phase of light. Moreover, this study demonstrated the reconfigurable capabilities of these metamolecules by showcasing dynamic beam steering and holographic wavefront reconstruction. In addition to incorporating ITO or graphene into metasurfaces, there are other approaches to realize electro-optical modulation. For example, Atwater’s group proposed a metasurface with multiple-quantum-well (MQW) structures [83], which can exhibit the quantum-confined Stark effect (QCSE) for high-speed modulation [92]. In Figure 3(f), the MQW elements can induce narrowband resonances with high quality factors, thus enhancing the optical modulation. By applying a voltage bias across the MQW structure, the appearance of QCSE results in a change in the refractive index. From the measurement results, the relative reflectance can achieve 270 % with a corresponding phase shift of ∼70°. Moreover, another critical feature of this work is that this MQW-based metasurface was grown monolithically so that this approach can be beneficial to the integration with other photonic devices. Considering the compatibility with the complementary metal-oxide-semiconductor (CMOS) process, doped semiconductors have become another option as metasurface materials. In Figure 3(g), Harris’s group fabricated a metasurface composed of doped Ge pillars with the coating of the gate oxide and Al-doped ZnO (AZO) [84]. It is noted that this combination makes the metasurface become a semiconductor-oxide-semiconductor (SOS) capacitor. Therefore, when an external voltage is applied to the metasurface, the carrier density in the Ge and AZO layers changes due to the field effect, which leads to the modulation of the effective refractive index for the metasurface. The reflectance spectra indicate that the shift of the resonance wavelength can go up to 240 nm in the mid-infrared when the voltage bias is tuned from −4 to 4 V. Moreover, the optical phase shift of the reflected light is estimated to achieve 270° under the optimal structural parameters of the Ge pillars. Mosallaei’s group introduced an innovative metasurface design featuring a zigzag pattern of elliptical silicon nanodisks, linked within each column by silicon nanobars serving as biasing lines [93]. It is noted that the elliptical nanodisks and biasing lines in the zigzag arrangement are divided vertically into three pairs of p−n junctions, thereby forming a multijunction p−n structure. Under an applied bias voltage, the electro-refraction induced by carrier accumulation in the multijunction p−n structures results in the electro-optical shift of the Huygens mode. A phase tuning of 240° can be achieved while maintaining an average transmission amplitude of 0.77.

### 2.4 Thermo-optically tunable metastructure nanophotonics and applications

**Figure 4:**
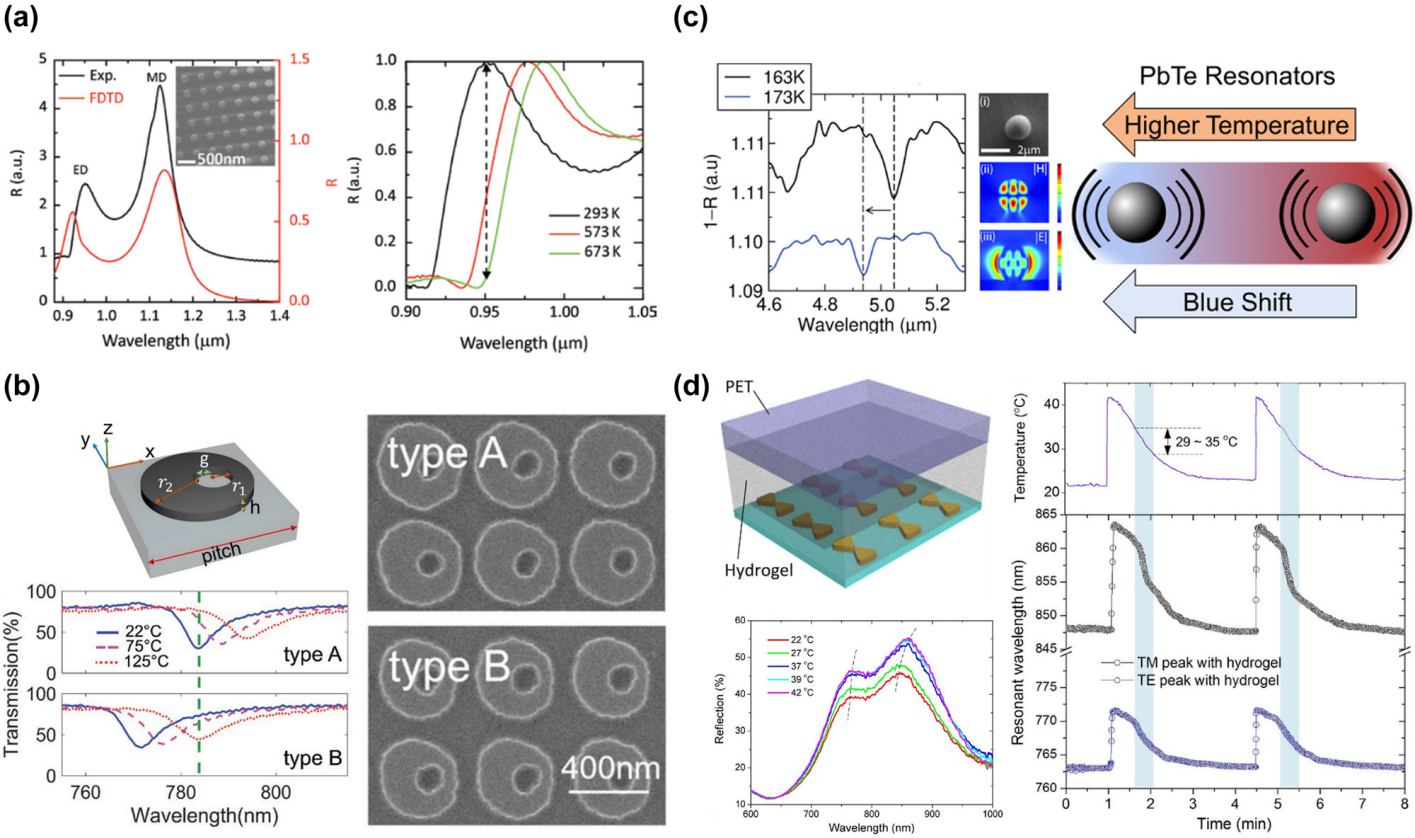
Tunable metastructures based on thermo-optical modulation. (a) Simulated and measured reflection spectra of a Si metasurface and the variation of reflection spectrum at different temperatures. Ref. [94] licensed under Creative Commons Attribution 4.0 License. (b) Schematic and SEM pictures of a Fano-resonance-based metasurface and the measured transmission spectra for two types of design at different temperatures. Reproduced with permission from ref. [96]. Copyright 2019 John Wiley and Sons. (c) Resonance wavelength shift under different temperatures with an SEM picture and simulation results for a PbTe meta-atom (left); working principle of a metasurface composed of PbTe meta-atoms (right). Reprinted (adapted) with permission from ref. [15]. Copyright 2017 American Chemical Society. (d) Schematic of a metasurface coated with a thermosensitive hydrogel layer and the reflection spectra with the temperature variation (left); evaluation of the thermal response time with different polarized light (right). Ref. [98] licensed under Creative Commons Attribution 4.0 License.

Thermal tuning is one of the typical methods to induce refractive index variation in tunable photonic devices. For instance, Schuller et al. investigated the thermal tunability for the commonly used semiconductors silicon and germanium in the infrared range [94]. By exciting the Mie resonance in a single meta-atom resonator, the thermos-optic coefficient (TOC) was found to be positive (d*n*/d*T* > 0) under low and intermediate temperatures but turns to negative at higher temperatures and longer wavelengths. To evaluate the performance of the thermal tuning in practical applications, a Si nanodisk array was designed and demonstrated as shown in Figure 4(a). The measured results indicate that the amplitude modulation could reach 13 dB when a 380 K temperature gradient was applied. Recently, some research works have attempted to employ the concept of Fano resonances in metasurface designs to induce narrowband resonances, which can be utilized for the applications of switches and filters [95]. In Figure 4(b), Rahmani et al. developed a Fano-resonance-based metasurface composed of silicon disks with a nonconcentric hole [96]. The off-center hole in this design can cause the interference between the electric dipole and the magnetic dipole, thus inducing the Fano resonance Thanks to the thermal-optical properties of silicon, the Fano resonance on the spectrum can be tuned by temperature. To demonstrate the modulation of the image contrast, two sets of silicon disks were applied on a Yin-Yang pattern to show the transmission contrast at different temperatures. The captured images exhibit a complete control of image contrast with the temperature change from 22 °C to 125 °C, and this modulation is reversible. Among thermoelectric materials [97], PbTe exhibits a high refractive index (*n* ∼ 6) and a large thermo-optic coefficient (TOC), which can enhance the capability for thermal-optical tuning. Due to these promising properties of PbTe, Schuller’s group proposed to employ PbTe meta-atoms in a metasurface to induce thermally tunable Mie resonances in the infrared (Figure 4(c)) [15]. From the experimental results under different temperatures, a single PbTe meta-atom was found to have larger resonance wavelength shifts in spectra at lower temperatures (80−293 K), resulting in higher TOC. Besides, the resonances can be shifted over one linewidth while the temperature is modulated by only ∼10 K. To further assess the performance of PbTe, a metasurface composed of PbTe meta-atoms was simulated, and the reflection spectrum shows that the resonance can achieve a high quality factor of 4500. In addition to thermoelectric materials, thermosensitive hydrogel coating is another promising method to realize thermal-optically tunable metasurfaces. In Figure 4(d), Dong et al. fabricated a gold bowtie nanoantenna array and the top surface was coated with a thermosensitive hydrogel layer to strengthen the response to the thermal variation [98]. When the environmental temperature changes, the hydrogel varies between hydrophobic and hydrophilic states, resulting in the change of the corresponding refractive index. With this index change, the surface plasmon resonances excited between the gold bowties can be modulated thermally. As the environmental temperature increases from 22 °C to 42 °C, the measured spectra show resonance shifts of 16.2 nm and 8 nm for the transverse-magnetic (TM) and transverse-electric (TE) polarized light, respectively. However, without hydrogel coating on the bowtie array, the resonance shifts are dropped to 3 nm and 1.6 nm for the TM and TE polarization, respectively. As for the modulation speed of this device, the response time toward the temperature change is found to be 250 ms and the switching time for reflectance is around 1 s.

### 2.5 Magneto-optically tunable metastructure nanophotonics and applications

**Figure 5:**
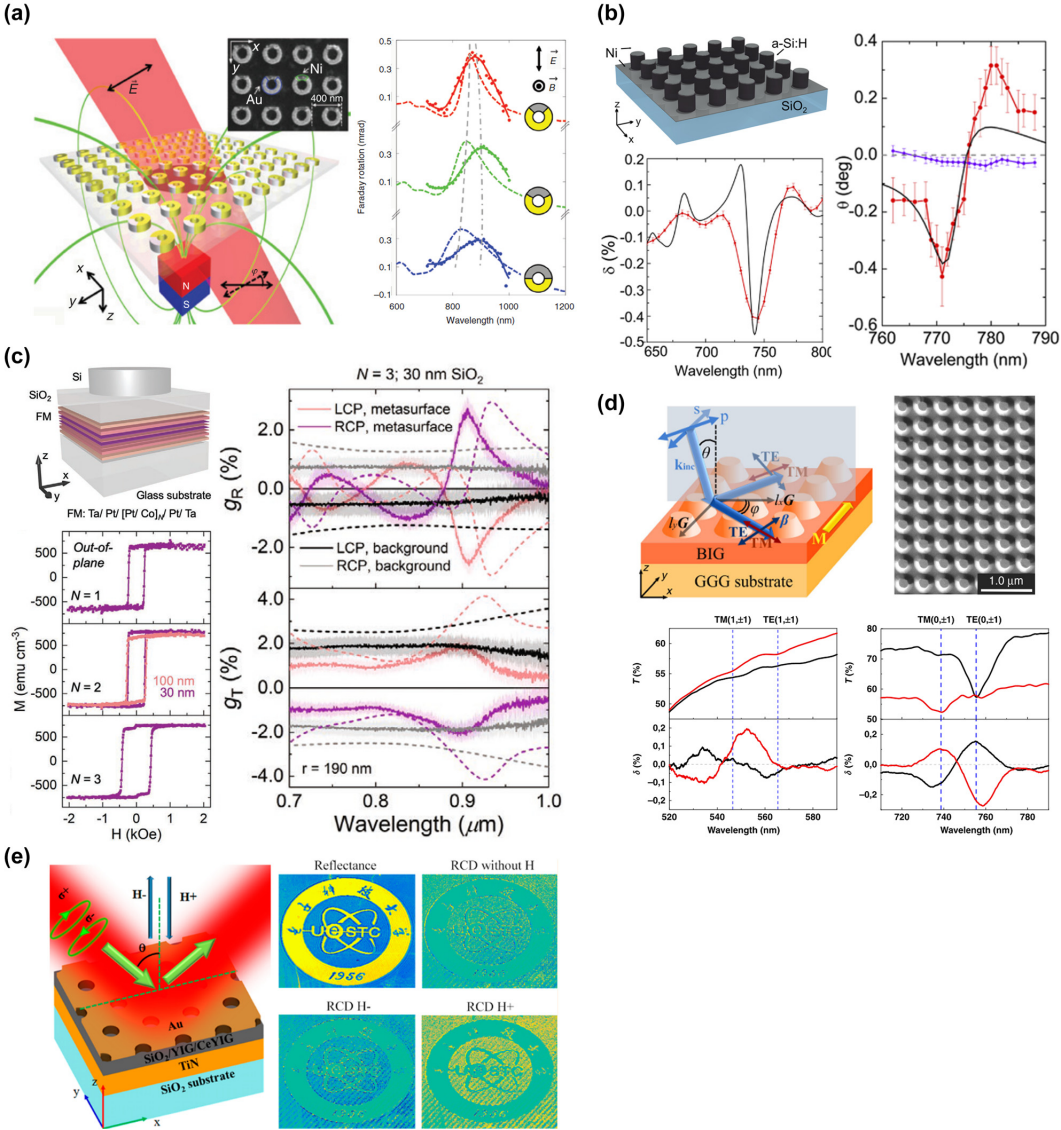
Tunable metastructures based on magneto-optical modulation. (a) Schematic and SEM image of a metasurface incorporating different ratios of gold and nickel to enable Faraday polarization rotation. Reproduced from ref. [99] with permission from De Gruyter. (b) Schematic of a hybrid Ni-Si metasurface and the experimental/numerical results for magneto-optical response and Faraday rotation spectra. Ref. [100] licensed under Creative Commons Attribution 4.0 License. (c) Schematic of a Si metasurface with [Pt/Co]_*N*_ multilayer stacks and the magnetic hysteresis loops for different *N* (left); measured and simulated dissymmetry of reflection and transmission spectra for circularly polarized light when *N* = 3 (right). Reproduced with permission from ref. [101]. Copyright 2020 John Wiley and Sons. (d) Schematic and SEM image of an all-dielectric magnetic metasurface made of BIG material and the measured spectra for transverse magnetophotonic intensity effect. Ref. [102] licensed under Creative Commons Attribution 4.0 License. (e) Working principle and demonstration of a magnetic metasurface with MIM cavity structure that enables the modulation of chiral imaging properties. Reprinted (adapted) with permission from ref. [103]. Copyright 2020 American Chemical Society.

Zheludev’s group presented a nanoring array incorporating gold and nickel to enable Faraday polarization rotation as presented in Figure 5(a) [99]. Nickel is a commonly used magnetized substance and a proper combination of nickel and gold can result in a strong magneto-optical (MO) response while maintaining a low dissipation loss. The nanoring structures with different nickel sector angles were fabricated and assessed for MO response. The measured Faraday spectra exhibit a maximal peak rotation of ∼0.41 mrad at 880 nm with 10 % transmittance when the nickel sector angle is 90°. The corresponding filling factor for nickel is ∼6 % and the calculated FOM is 2.16 × 10^−3^, which is better than the result for a nickel film with the same thickness (FOM ∼ 0.33 × 10^−3^). In addition to the bimetal structure, Fedyanin’s group hybridized the silicon nanodisks and thin nickel film to enhance the magneto-optical response as shown in Figure 5(b) [100]. In the measured transmission spectrum, two resonance dips were found due to the excitation of the magnetic dipole and the electric dipole. To assess the MO enhancement of this hybrid structure, two experimental setups were built to observe the Faraday effect and the Voigt effect. The measurement results show that the Faraday rotation angle can achieve 0.8° and the maximum magneto-optical response is ∼0.5 %. It is noted that the thickness of nickel applied on this metasurface is only 5 nm, which exhibits a significant Faraday rotation of 160°/µm. Besides the above nickel-based structures, Dionne et al. developed ferromagnetic [Pt/Co]_*N*_ multilayer stacks to enhance the MO effects with circularly polarized light [101]. As shown in Figure 5(c), the multilayer films are composed of Ta/Pt/[Pt/Co]_*N*_/Pt/Ta with a SiO_2_ layer and a Si nanodisk array on the top. The magnetic hysteresis measurements show that the saturation magnetization and the uniaxial anisotropy constants increase with increasing *N*. The dielectric metasurface can lead to the excitement of the Mie-type dipolar resonances, and the simulation exhibits a significant increase in the local electric field rotation inside the ferromagnetic films. From the experimental results, the reflection and transmission of circularly polarized light can reach the dissymmetry of 2 %. Moreover, the calculated differential absorption of circularly polarized light becomes higher compared to that of the unpatterned films, which indicates the proposed. structure allows better performance for the light-assisted magnetic switching. Although magnetic metals like Ni and Co mentioned above can demonstrate remarkable MO effects, the high absorption loss may hinder their practical applications. In Figure 5(d), Belotelov’s group fabricated an all-dielectric magnetic metasurface that can modulate both p-polarized and s-polarized light [102]. The metasurface is made of a bismuth-substituted iron-garnet (BIG) nanopillar array on an iron-garnet film. Compared to magnetic metals, BIG allows metasurfaces to have higher transmission and resonances with higher quality factors while exhibiting promising MO properties. In the measurement results, the transmittance of the metasurface is higher than 50 % and the quality factor of a guided-mode resonance can achieve up to 110 with a transmission depth of 30 %. It is found that when the guided mode propagates along an angle with respect to the incidence plane, both TE and TM polarized modes can be excited at the same time, which strengthens the MO modulation of the light intensity. To enhance the chirality modulation for magnetic metasurfaces, Bi’s group integrated the magnetic oxide material Ce_1_Y_2_Fe_5_O_12_ (Ce: YIG) with gold nanoholes and TiN thin film to build a metal-dielectric-metal (MIM) cavity structure as indicated in Figure 5(e) [103]. While applying magnetic fields on the metasurface from −0.6 ± 0.2° to +1.9 ± 0.1° with 950 nm wavelength circularly polarized light, the amplitude modulation of circular dichroism can go up to ∼2.5° and a sign reversal was found under reversing magnetic field. It is noted that the nanoholes on the gold layer were fabricated using a polystyrene (PS) sphere self-assembly. As a result, a 2 mm by 2 mm metasurface was fabricated to demonstrate the modulation of chiral imaging properties, which exhibit the potential for tunable chiroptical applications.

**Table 1:** Tunable nanophotonic devices.

Type	Tuning mechanism	Refractive index tuning range	Tuning speed	Non-volatility	References
Metastructure nanophotonics	Phase change	∼2.0	0.2 Hz	No	[27]
		4.8	Not reported	No	[28]
		∼1.32	5 Hz	No	[33]
		1–1.5	Not reported	No	[34]
		∼0.5	Not reported	No	[35]
		∼0.15	Not reported	No	[44]
		0.39	65 ms (on)/40 ms (off)	No	[36]
		∼0.2	Not reported	No	[37]
		0.5	1 kHz	No	[46]
		0.21	1 kHz	No	[47]
		0.184	Not reported	No	[48]
		∼1.0	Not reported	Yes	[49]
		∼2.7	Not reported	Yes	[50]
		3.0	Not reported	Yes	[51]
		∼0.3	Not reported	Yes	[52]
		∼1.6	Not reported	Yes	[53]
		∼2.5	Not reported	Yes	[54]
		∼2.9	Not reported	Yes	[55]
		2.0–2.3	∼190 ns (C)/∼100 ns (A)	Yes	[56]
		∼2.8	1 kHz	Yes	[38]
		∼2.0	Not reported	Yes	[58]
		2.93	Not reported	Yes	[59]
		∼0.73	∼21 µs (C)/∼550 ns (A)	Yes	[39]
		∼2.5	∼300 µs (C)/∼650 ns (A)	Yes	[60]
		0.4	Not reported	Yes	[61]
		1.76	∼0.4 Hz	Yes	[63]
		∼1.6	20 ms (C)/13 µs (A)	Yes	[64]
		1.76	1 ms (C)/1 µs (A)	Yes	[40]
		1.25–1.5	Not reported	Yes	[41]
	Mechanical	NA	∼4 kHz	No	[66]
			∼50 kHz		[67]
			100 Hz		[68]
	Electro-optical	∼1.0	125 kHz	No	[72]
		Not reported	10 MHz		[74]
		Not reported	600		[75]
		∼1.68	Not reported		[76]
		∼1.08	Not reported		[79]
		∼1.35	Not reported		[80]
		Not reported	40 MHz		[88]
		Not reported	7.2 GHz		[81]
		0.02	1 MHz		[83]
		Not reported	100 kHz		[84]
	Thermo-optical	0.1–0.15	Not reported	No	[94]
		0.7	2.6 μs		[15]
		0.065	250 ms		[98]
	Magneto-optical	∼1.91 × 10^−3^	Not reported	No	[99]
		∼0.68			[100]
		∼0.54			[101]

C, crystallization; A, amorphization.

## 3 Summary

We reviewed the tunable nanophotonic metastructures based on various tuning mechanisms including phase-change-mechanisms, thermo-optical, electro-optical (e.g. Pockels and Kerr effects), magneto-optical, ionic-optical, piezo-optical, mechano-optical (deformation in MEMS or NEMS), and phase-change mechanisms. A summary is shown in Table 1. Such mechanisms can alter the real and/or imaginary parts of the optical susceptibility tensors, leading to tuning of the optical characteristics. In particular, tunable nanophotonic metastructures with relatively large tuning strengths (e.g. large changes in the refractive index) can lead to particularly useful device applications including imaging, computing, communications, and sensing. Among the reviewed tunable nanophotonic metastructures, some of the phase-change-mechanisms offer relatively large index change magnitude while offering non-volatility. In particular, GSST and VO_2_ materials are popular for this reason. Mechanically tunable nanophotonic metastructures offer relatively small changes in the optical losses while offering large index changes. Electro-optically tunable nanophotonic metastructures offer relatively fast tuning speeds while achieving relatively small index changes. Thermo-optically tunable nanophotonic metastructures offer nearly zero changes in optical losses while realizing modest changes in optical index at the expense of relatively large power consumption. Magneto-optically tunable nanophotonic metastructures offer non-reciprocal optical index changes that can be induced by changing the magnetic field strengths or directions.

While tunable nanophotonic metastructures are attractive for a wide range of applications, there are two main challenges for their practical and scalable deployments. First is the material compatibility with tunable materials such as GSST, VO_2_, PbTe, etc., with common fabrication processflows in commercial foundries. Second is nanofabrication methods for these structures are dominated by methods such as e-beam lithography on a relatively small areas of full wafers. There challenges can possibly overcome in the new trends of silicon photonics where foundries are adopting nanoscale CMOS fabrication techniques for wafer-scale silicon photonic fabrication wherein nanoscale metastructures can be lithographied and patterned by projection lithography. Recent trends at the foundries have shown postfabrication methods applied to incorporate new materials and structures that are not fully compatible with CMOS in the Back-End-Of-the-Line (BEOL) processes such as eMRAMs [104], PRAMs, or optical MEMS [105], or III-V lasers [106]. Hence the strong application drivers of tunable nanophotonic metastructures may soon push such structures to be incorporated into scalable manufacturing techniques on advanced (<14 nm) CMOS manufacturing platforms.
